# Balloon Aortic Valvuloplasty for Severe Aortic Stenosis as Rescue or Bridge Therapy

**DOI:** 10.3390/jcm10204657

**Published:** 2021-10-11

**Authors:** Pawel Kleczynski, Aleksandra Kulbat, Piotr Brzychczy, Artur Dziewierz, Jaroslaw Trebacz, Maciej Stapor, Danuta Sorysz, Lukasz Rzeszutko, Stanislaw Bartus, Dariusz Dudek, Jacek Legutko

**Affiliations:** 1 Department of Interventional Cardiology, Institute of Cardiology, Jagiellonian University Medical College, John Paul II Hospital, Pradnicka 80 Street, 31-202 Krakow, Poland; jartrebacz@gmail.com (J.T.); maciej.stapor@gmail.com (M.S.); jacek.legutko@uj.edu.pl (J.L.); 2 Students’ Scientific Group at the Department of Interventional Cardiology, Jagiellonian University Medical College, John Paul II Hospital, Pradnicka 80 Street, 31-202 Krakow, Poland; alexandra.kulbat@gmail.com (A.K.); piotrbrzy@gmail.com (P.B.); 3 2nd Department of Cardiology, Institute of Cardiology, Jagiellonian University Medical College, University Hospital, Jakubowskiego 2 Street, 30-688 Krakow, Poland; adziewierz@gmail.com (A.D.); dtsorysz@op.pl (D.S.); lrzeszutko@cathlab.krakow.pl (L.R.); mbbartus@cyf-kr.edu.pl (S.B.); mcdudek@cyfronet.pl (D.D.)

**Keywords:** aortic stenosis, balloon aortic valvuloplasty, bridge therapy, destination therapy, heart failure

## Abstract

The study aimed to assess procedural complications, patient flow and clinical outcomes after balloon aortic valvuloplasty (BAV) as rescue or bridge therapy, based on data from our registry. A total of 382 BAVs in 374 patients was performed. The main primary indication for BAV was a bridge for TAVI (*n =* 185, 49.4%). Other indications included a bridge for AVR (*n =* 26, 6.9%) and rescue procedure in hemodynamically unstable patients (*n =* 139, 37.2%). The mortality rate at 30 days, 6 and 12 months was 10.4%, 21.6%, 28.3%, respectively. In rescue patients, the death rate raised to 66.9% at 12 months. A significant improvement in symptoms was confirmed after BAV, after 30 days, 6 months, and in survivors after 1 year (*p* < 0.05 for all). Independent predictors of 12-month mortality were baseline STS score [HR (95% CI) 1.42 (1.34 to 2.88), *p* < 0.0001], baseline LVEF <20% [HR (95% CI) 1.89 (1.55–2.83), *p* < 0.0001] and LVEF <30% at 1 month [HR (95% CI) 1.97 (1.62–3.67), *p* < 0.0001] adjusted for age/gender. In everyday clinical practice in the TAVI era, there are still clinical indications to BAV a standalone procedure as a bridge to surgery, TAVI or for urgent high risk non-cardiac surgical procedures. Patients may improve clinically after BAV with LV function recovery, allowing to perform final therapy, within limited time window, for severe AS which ameliorates long-term outcomes. On the other hand, in patients for whom an isolated BAV becomes a destination therapy, prognosis is extremely poor.

## 1. Introduction

At present, management of severe aortic valve stenosis (AS) offers surgical or endovascular therapy depending on a patients’ risk profile and severity of clinical symptoms [1,2]. Endovascular treatment includes transcatheter aortic valve implantation (TAVI) or balloon aortic valvuloplasty (BAV). There are also patients who are too sick to benefit from invasive treatment and thus scheduled to conservative therapy with the worst prognosis. Balloon aortic valvuloplasty has gained importance in recent years, especially in patients who were recognized as in not optimal clinical condition for any definitive treatment due to severe comorbidities. Baloon valvuloplasty can either serve as a standalone palliative procedure performed in haemodynamicaly unstable patients or as a bridge to final therapy [1,2,3]. Furthermore, BAV allows AS patients to undergo an urgent non-cardiac surgery with its good immediate hemodynamic result [3,4]. On the contrary to TAVI, clinical and hemodynamic outcomes of BAV were shown to be relatively poor with longer follow-up period, and sometimes the procedure needs to be repeated [4,5,6,7,8]. Due to relatively low access to TAVI, BAV is still a reasonable procedure in developing countries, but such an approach remains rather controversial in light of current ESC guidelines [2]. Thus, we aimed to assess patient flow, procedural complications and clinical outcomes after BAV as rescue or bridge therapy in patients with severe symptomatic AS.

## 2. Materials and Methods

In current study, the data of all consecutive patients with severe symptomatic AS with an aortic valve area (AVA) < 0.7 cm^2^ (indexed AVA < 0.5 cm^2^/m^2^ body surface area) and/or mean transaortic gradient ≥40 mmHg who underwent BAV between December 2008 and May 2021 at two tertiary university centers, were included. Left ventricle ejection fraction (LVEF) was assessed with transthoracic echocardiography (TTE) using the modified Simpson’s method of discs, acquiring LV volumes from apical 4- and 2-chamber view. The study was conducted as a two center, retrospective registry. Patients were carefully examined to assess the operative risk, comorbidities and procedural feasibility. Patient screening and selection were performed by a multidisciplinary ‘Heart Team’. The institutional ethical board was informed and approved our study. The procedure was preceded by coronary angiography in the vast majority of cases and guided by TTE and fluoroscopy. Femoral access was used, starting with a 6F sheath and the exchanged to destination sheath depending on the balloon size. Anticoagulation was achieved with unfractionated heparin with activated clotting time of 250 to 300 s. Balloons from Osypka Medical Inc. (Berlin, Germany) were used in most cases. Balloon sizes were chosen depending on a minimal annulus diameter measured in TTE or based on CT scans, if available. The exact positioning of the balloon during inflation was obtained with rapid ventricular pacing from either the 0.035” ultra-stiff guidewire inserted into the left ventricle (LV) or the temporary pacemaker inserted into the right ventricle (RV) (via a 6 or 7 Fr venous sheath) [9,10]. The number of balloon inflations was left to the operator’s discretion. Successful procedure was described as transaortic gradient drop of more than 30% compared to baseline. Vascular access was closed with manual compression or an Angio-Seal device (Terumo, Tokio, Japan). Baseline clinical, echocardiographic and procedural data, as well as complication rates, were analyzed.

### Statistical Analysis

Continuous variables were expressed as a median (interquartile range) and categorical variables were expressed as a number (percentage). Continuous variables were compared by t-test for dependent samples when normally distributed or by Wilcoxon signed-rank test when not normally distributed. Categorical variables were compared by Pearson’s v2 test and Fisher’s exact test. The Pearson rank correlation coefficient for normally distributed variables or Spearman’s rank correlation coefficient for not normally distributed variables were calculated to test the association between two variables. Cox regression models for all-cause mortality were constructed to identify independent predictors of survival. Age, sex, hypertension, coronary artery disease, diabetes, atrial fibrillation, cerebrovascular incident, pulmonary disease, chronic kidney disease, STS score, LVEF < 20% at baseline, LVEF < 30% at 1 month, which were identified a priori as clinically relevant and included in the model. The significance level was set at *p* < 0.05. Statistical analysis was performed using IBM SPSS Statistics for Windows, Version 25.0 (IBM Corporation, Armonk, NY, USA). 

## 3. Results

**Figure 1:**
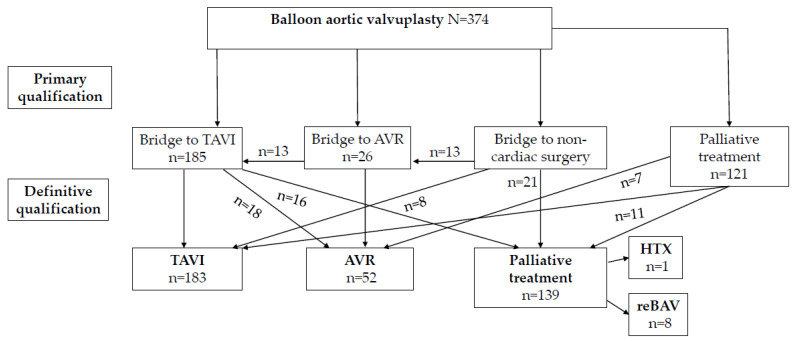
Flowchart of patients undergoing balloon aortic valvuloplasty as a bridge or final therapy. AVR—aortic valve replacement; BAV—balloon aortic valvuloplasty; HTX—heart transplantation; TAVI—transcatheter aortic valve implantation.

A total of 382 BAVs in 374 patients was performed. Procedural success was present in 94.6% of patients. Repeat BAV was performed in 8 patients. The main primary indication for BAV was a bridge for TAVI (*n =* 185, 49.4%). Other indications included a primary bridge for aortic valve implantation (AVR, *n =* 26, 6.9%) and palliative treatment (*n =* 139, 37.2%). Twenty-two patients (5.8%) underwent BAV in the course of cardiogenic shock and in 42 (11.2%) patients BAV was performed before urgent non-cardiac surgery. Finally, 183 (48.9%) of patients after BAV underwent TAVI and 52 (13.9%) patients underwent AVR during follow-up. In some patients who were primarily qualified to TAVI or AVR as described above, the final qualification has changed during the follow-up period (Figure 1). The median follow-up period was 686 days and ranged from 103 to 1245 days.

### 3.1. Baseline Characteristics

The median age of the enrolled population was 84 years, with a higher prevalence of females (53%). All patients presented symptoms of NYHA class III or IV. The median STS score was 10.1% and logistic Euroscore II was 7.4. Clinical data are presented in Table 1.

### 3.2. Procedural Data

Concomitant coronary angiography with BAV was performed in 355 (94.9%) patients and concomitant PCI was performed in 81 (21.6%) patients. Only severe coronary lesions with >70% stenosis were treated in ostial or proximal segments of major epicardial arteries. The median balloon size was 22 mm [IQR 18–24.5 mm]. Wire LV pacing was used in 196 patients (52.4%). Eight patients underwent repeated procedures within a median of 189 days. The remaining procedural data are shown in Table 2.

### 3.3. Echocardiographic Data

All echocardiographic baseline and follow-up data are showed in Table 3. There were 8 severe aortic regurgitations (AR) after BAV and 4 patients were successfully treated with TAVI, 2 of them underwent successful AVR. The remaining 2 severe ARs resulted in intrahospital death. Echocardiographic examinations performed after BAV and at 30 days, 6 and 12 months showed that AVA was higher up to 6 months, (*p* < 0.05 for all), but the mean transaortic gradient did not show significant differences after 6 months compared to values directly after BAV. Interestingly, in 93 (24.6%) patients with impaired left ventricular function (LVEF <30%) a significant improvement of LVEF (median 18 %) after 30 days (*p* = 0.025) was observed and it was sustained after 6 months (*p* = 0.034). A response to BAV (improvement of LVEF) was observed in 235 patients (63.8%), without any progress of LVEF impairment due to BAV. These circumstances allowed patients to be requalified to definitive therapy, TAVI or AVR. Improvement of LVEF correlated with a change of AVA (r = 0.6, *p* < 0.0001) and mean transaortic gradient (r = 0.72, *p* < 0.0001). 

### 3.4. Complications

Detailed data on complications rate are presented in Table 4. Major complications occurred in 97 patients: (1) intraprocedural death (*n =* 9, 2.4%; 3 fatal tamponades, 1 fatal complete atrioventricular block, 5 cardiogenic shocks due to severe AS), (2) cardiac tamponade (*n =* 9, 2.4%), (3) severe AR (*n =* 8, 2.1%), (4) severe cardiac arrhythmias (*n =* 18, 4.8%), (5) cerebrovascular incident (*n =* 6, 1.6%), (6) permanent pacemaker implantation (*n =* 3, 0.8%), (6) need for red blood cells transfusion (*n =* 31, 8.2%), (7) urgent cardiac surgery (*n =* 13, 3.5%). Fatal annulus rupture was noted in 1 patient. Of 9 tamponades, 3 resulted in intraprocedural death, 2 with conversion to AVR and 3 were successfully treated with pericardiocentesis. Cardiac tamponade was caused by the temporary electrode placed in the RV in 2 cases confirmed by cardiac surgeons. Two cases were caused by the PM inserted into the RV, based on echo images. One tamponade resulted from annulus rupture. In one case the cause of tamponade remained unclear. Complete atrioventricular block despite stimulation was the cause of one intraprocedural death. Permanent pacemaker implantations were due to complete atrioventricular block occurring directly after BAV. Vascular access site complications occurred in 47 patients (12.5%). Arterial pseudoaneurysms were successfully treated with either manual compression or direct thrombin injection. 

### 3.5. Outcomes

A significant improvement in symptoms (NYHA and CCS) was confirmed after BAV, after 30 days, 6 months and in survivors after 1 year (*p* < 0.05 for all). 

In-hospital mortality was 5.1%. In addition to 9 intraprocedural deaths described previously, 4 patients died due to acute respiratory failure despite mechanical ventilation and 6 more had a sudden cardiac arrest (fatal ventricular tachycardia or fibrillation) in the intensive care unit after index procedure. 

All-cause mortality rate at 30 days, 6 and 12 months was 10.4%, 21.6%, 28.3%, respectively. However, analyzing the death rate for palliative patients, the rate raised to 66.9% at 12 months (Table 5). Deaths in the palliative group were defined as cardiovascular in 72 (51.8%) cases (recurrent heart failure, sudden cardiac death, major stroke, pulmonary embolism or unknown reason) and non-cardiovascular in 21 (15.1%) cases (cancer, major gastrointestinal bleeding). 

Patients treated with BAV in the course of cardiogenic shock had the worst prognosis with 22.7% intraprocedural mortality and 81.9% in-hospital mortality. 30-day mortality was 100% in this group. 

Of 121 palliative patients, one patient was qualified for heart transplantation, 11 have changed qualification for TAVI and subsequently underwent TAVI; 7 were qualified for AVR and subsequently underwent AVR (Figure 1). Eight patients underwent repeat BAV. Among 139 patients in the destination therapy group, we found significant differences in survivors compared to fatal cases in terms of: age (82.2 vs. 86.8 years, *p* = 0.001), logistic EuroSCORE II (7.2% vs. 9.5%, *p* = 0.004), STS score (10.3 vs. 12.8, *p* = 0.002), baseline LVEF (45.3% vs. 31.4%, *p* < 0.001), respectively. 

In the subgroup of patients initially bridged for TAVI (Figure 1): 185 successfully underwent TAVI, 24 died before the intended procedure and 16 were excluded due to progressive dementia, mitral stenosis, malignancy, or severe impairment of mobility. Eighteen patients who were to undergo TAVI were switched to AVR because of concomitant severe tricuspid regurgitation, large aortic annulus and improvement of LVEF after BAV. Seven patients were bridged for AVR. All patients bridged to noncardiac surgery successfully underwent their intended procedures, in 13 patients AVR and in 8 - TAVI was performed after noncardiac procedure. The rest of assessed patients remained in the palliative treatment group. 

In multivariable logistic regression analysis, we identified following independent predictors of 12-month all-cause mortality (Table 6): baseline STS score [HR (95% CI) 1.25 (1.08 to 1.94), *p* = 0.001], baseline LVEF < 20% [HR (95% CI) 1.65 (1.04–2.67), *p* = 0.02] and LVEF <30% at 1 month [HR (95% CI) 1.87 (1.35–3.43), *p* = 0.001]. Independent predictors of 12-month mortality were baseline STS score [HR (95% CI) 1.42 (1.34 to 2.88), *p* < 0.0001], baseline LVEF < 20% [HR (95% CI) 1.89 (1.55–2.83), *p* < 0.0001] and LVEF < 30% at 1 month [HR (95% CI) 1.97 (1.62–3.67), *p* < 0.0001] adjusted for age/gender.

## 4. Discussion

Our study of 374 patients shows that balloon aortic valvuloplasty is a relatively safe and crucial procedure in patients who are at first too sick to be scheduled for TAVI or AVR. Therefore, bridging therapy is necessary to change the primary qualification because of left ventricle function improvement. Moreover, BAV may be an option for extremely comorbid high-risk patients as a palliative intervention for symptom relief, despite very high 12-month all-cause mortality of almost 67%. Left ventricle ejection fraction at baseline and at 1 month as well as baseline STS score were identified as independent predictors of 12-month all-cause mortality. Balloon valvuloplasty may also be important for patients with severe AS who must undergo an urgent non-cardiac surgery. 

Hemodynamic results of BAV included an increase of AVA, a decrease of maximal and median transaortic gradient immediately after the procedure what has been presented previously [11,12,13,14,15,16,17]. The effect of the procedure was sustained for 1 month and started to diminish gradually at 6 months, however not achieving preprocedural values of AVA, LVEF and transaortic gradients. Nonetheless, at 12 months, the effects of BAV in survivors in the destination therapy group were abated and were similar to baseline values. This highlights the recurrence of AS severity and symptoms with longer time period from BAV. Moreover, a 6-month period following BAV seems to be crucial for bridging to final treatment (TAVI or even AVR) for this subset of patients. Left ventricle ejection fraction recovery after BAV seems to be a turning point for the final treatment pathway, either conservative treatment burdened with high mortality rate or interventional/surgical treatment improving outcomes. Despite favorable acute results, long-term mortality remained high, especially in patients in destination treatment cohort. Also, we noted a relevant rate (ca. 15%) of non-cardiac death for patients after BAV, which may be related to a selection bias wherein those patients are excluded from a more definite treatment due to many comorbidities.

Procedure-related complications rate was similar to that showed in previous studies [3,8,9,13,15,16]. A high rate of vascular complications, up to 12.5%, was reported and mostly related to the use of large arterial sheaths (8–10 F) and peripheral arterial disease. Also, additional venous sheath insertion (in 47.3% of cases) might have contributed to access-site related complications. On the other hand, these rates were lower than reported for TAVI [18]. In the case of peripheral artery disease affecting both iliac arteries, transradial or transbrachial access is possible with the use of one or two balloons [19,20]. Periprocedural deaths were, in fact, limited to patients with hemodynamic instability/cardiogenic shock before the procedure. Once periprocedural death resulted from annulus rupture. In contrast to previous reports, we did not observe myocardial infarction during or after BAV procedure [6,16]. Balloon rupture during BAV may occur due to bulky calcium load from the diseased valve, which was the case in 6.1% of cases in our study. However, none of these resulted in the cerebrovascular incidents. After rupture, removing the balloon with the delivery system is more challenging, especially if a boundary size of the arterial sheath, compatible with the balloon’s diameter, was used [21].

Our observations are in line with data shown in other studies showing reprise of BAV in the era of TAVI [3,4,8,9,11,12,13,15,16,20,22]. When BAV is used as a bridge, it gives some time and opportunity to improve the clinical and hemodynamic response among treated patients with severe AS not being appropriate candidates for final treatment at that time point. The hemodynamic effects of BAV with subsequent LVEF improvement can be a turning point in decision and planning further treatment with TAVI or AVR, especially when serious comorbidities contribute to overall risk profile, extreme frailty or very low ejection fraction affect outcomes seriously [23,24]. In the natural history of conservative course of severe AS during long-term follow-up more significant decrease in AVA and increase of pulmonary artery systolic pressure correlates with a lower reduction of LVEF [25]. This fact may suggest that LV contractility remains proofed longer to unfavorable hemodynamics caused by deteriorating valve disease. On the other hand, a small improvement in AVA after BAV could impact LVEF recovery as presumably, it may be more sensitive to any decrease in afterload. Summing up, current study provides additional evidence for possible improvement of initially depressed LVEF after BAV, what has also been postulated previously [26,27]. Patients become better candidates for TAVI/AVR and this may highlight the actual importance of BAV. However, considering the stepwise and consistent deterioration of valve parameters and the persistent high risk of death, a “watchful waiting strategy” should be preferred over routine follow-up after BAV. Moreover, LVEF >40% at 1 month might be helpful guidance for the decision about final therapy, which should be implemented at the longest of 6 months after BAV [28]. Otherwise, the prognosis would be dramatically worsened, as showed in the palliative group in our study. 

Balloon aortic valvuloplasty has also been investigated in patients with severe AS requiring urgent non-cardiac surgery [4,29,30,31,32,33], but no clear evidence has been shown so far, and studies showed disturbing results. In a study by Debry at al., the authors concluded that patients with severe AS managed conservatively before urgent non-cardiac surgery are at high risk of events and a systematic invasive strategy using BAV does not significantly improve clinical outcomes [4]. However, contrary to our results, patients enrolled had higher LVEF (56.6% in the invasive arm and 59.2% in the conservative arm) [4]. Current ESC guidelines for the treatment of valvular heart disease allow BAV to be performed in such patients with class IIb recommendation [2]. 

The role of cardiac rehabilitation in patients undergoing BAV remains unknown in contrast to the TAVI population [34,35,36]. Physical exercise conditioning may be, however, limited in these patients due to the profile of comorbidities, frailty and poorer LV function compared to TAVI patients. Nonetheless, close watchful waiting after BAV within an outpatient cardiac rehabilitation program may be beneficial, especially in terms of deterioration of the AS.

### Study Limitations

Current study has several limitations which are inherent to the non-randomized design. The study findings were derived from observational analyses, which are subject to well-known limitations. We could not exclude a residual bias related to the age of patients as well as other patient’s characteristics. 

## 5. Conclusions

In everyday clinical practice in the TAVI era, there are still clinical indications to BAV a standalone procedure as a bridge to surgery, TAVI or for urgent high risk non-cardiac surgical procedures. Patients may improve clinically after BAV with LV function recovery, allowing to perform final therapy, within a limited time window, for severe AS, which ameliorates long-term outcomes. On the other hand, in patients for whom an isolated BAV becomes a destination therapy, prognosis is extremely poor.

## Figures and Tables

**Table 1:** Baseline clinical characteristics.

	All (*n =* 374)
Age, median (IQR) (years)	84 (81.3–89.5)
Men, *n* (%)	176 (47.0)
Body mass index, median (IQR) (kg/m^2^)	24.7 (22.4–28.4)
Glomerular filtration rate, median (IQR) (mL/min/1.73 m^2^)	48 (37.5–75.2)
CCS class, *n* (%) I + II III IV	23 (6.1) 289 (77.3) 62 (16.6)
NYHA class, *n* (%)	
I + II	0
III	228 (60.9)
IV	146 (39.0)
Coronary artery disease, *n* (%)	324 (79.1)
Arterial hypertension, *n* (%)	192 (86.6)
Diabetes mellitus, *n* (%)	158 (42.2)
Atrial fibrillation, *n* (%)	103 (27.5)
History of myocardial infarction, *n* (%)	89 (23.8)
History of percutaneous coronary intervention, *n* (%)	106 (28.3)
History of coronary artery bypass grafting, *n* (%)	72 (19.2)
Chronic obstructive pulmonary disease, *n* (%)	79 (21.1)
Peripheral artery disease, *n* (%)	83 (22.1)
Stroke/transient ischemic attack, *n* (%)	49 (13.1)
Syncope, *n* (%)	57 (15.2)
Previous heart failure deterioration, *n* (%)	226 (60.4)
Cardiogenic shock, *n* (%)	22 (5.8)
Previous pacemaker, *n* (%)	43 (11.4)
Neoplasm, *n* (%)	41 (10.9)
Previous radiotherapy, *n* (%)	29 (7.7)
Porcelain aorta, *n* (%)	19 (5.0)
Logistic EuroSCORE II (%), median (IQR)	7.8 (5.6–14.2)
The Society of Thoracic Surgeons score (%), median (IQR)	11.1 (8.1–13.9)

**Table 2:** Procedural data.

	All (*n* = 374)
Concomitant coronary angiography, *n* (%)	355 (94.9)
Concomitant PCI, *n* (%)	81 (21.6)
Size of femoral arterial sheath, median (IQR) (Fr)	9 (8.0–10.0)
Size of femoral venous sheath if used, median (IQR) (Fr)	6 (6.0–7.0)
Unfractionated heparin dose, median (IQR) (units)	5000 (4000.0–6500.0)
Wire pacing, *n* (%)	196 (52.4)
Number of inflations, median (IQR)	1 (1–3)
Vascular closure device, *n* (%)	215 (57.4)
Manual compression after sheath(s) removal, *n* (%)	159 (42.5)
Balloon size, median (IQR) (mm)	22 (18–24.5)
Radiation dose (BAV alone), median (IQR) (Gy)	0.26 (0.15–0.45)
Contrast media volume (BAV alone), median (IQR) (mL)	10 (5.0–24.0)
Fluoroscopy time (BAV alone), median (IQR) (min)	7.4 (5.2–15.4)
Duration (BAV alone), median (IQR) (min)	26 (17.9–35.5)

BAV—balloon aortic valvuloplasty; PCI—percutaneous coronary intervention.

**Table 3:** Echocardiografic data.

	Baseline (*n* = 374)	After BAV (*n =* 365)	30 Days (*n =* 335)	6 Months (*n =* 293)	12 Months (*n =* 46) ^
Maximal transaortic gradient, median (IQR) (mmHg)	93.8 (81.2–104.82)	64 (47.2–73.5) *	67 (48.4–76.4) *	72.5 (55.3–85.3) *#	86.3 (58.6–97.4) #
Mean transaortic gradient, median (IQR) (mmHg)	41.1 (40.4–55.2)	31.3 (21.4–38.2) *	32.5 (22.4–39.4) *	38.4 (30.3–49.6) *#	40.6 (39.5–54.7) #
Aortic valve area, median (IQR) (cm^2^)	0.52 (0.42–0.61)	0.79 (0.65–0.92)	0.77 (0.66–0.90) *	0.71 (0.63–0.88) *#	0.53 (0.44–0.63) #
Left ventricle ejection fraction, median (IQR) (%)	41.2 (33.5–52.0)	44.2 (38.5–54.8)	48.2 (42.6–58.3) *#	46.7 (40.1–55.3)	42.9 (39.1–53.1) #
Right ventricular systolic pressure, median (IQR) (mm Hg)	53 (36.0–68.5)	45.2 (32.2–56.2) *	46.4 (33.2–57.6) *	49.3(35.2–62.2) #	52 (37.1–65.2) *#
Aortic regurgitation None/trivial, *n* (%) Mild, *n* (%) Moderate, *n* (%) Severe, *n* (%)	145 (38.7) 157 (41.9) 52 (13.9) 0 (0.0)	93 (24.8) 183 (48.9) 82 (21.9) * 8 (2.1)	86 (22.9) 191 (51.0) * 75 (20.0) * 0 (0.0)	105 (28.0) 206 (55.0) * 67 (17.9) 0 (0.0)	18 (39.1) 12 (26.0) 16 (34.7) 0 (0.0)

* *p* < 0.05 compared with baseline, # *p* < 0.05 compared after BAV. ^ outcomes of the final palliative group not qualified to TAVI or AVR.

**Table 4:** Complications.

	All (*n* = 374)
Severe aortic regurgitation after BAV, *n* (%)	8 (2.1)
Balloon rupture, *n* (%)	23 (6.1)
Cardiac tamponade, *n* (%)	9 (2.4)
Severe cardiac arrythmias, *n* (%)	18 (4.8)
Cerebrovascular incident, *n* (%)	6 (1.6)
Vascular access site complications, *n* (%)	47 (12.5)
hematoma, *n* (%)	18 (4.8)
pseudoaneurysm, *n* (%)	17 (4.5)
arteriovenous fistula, *n* (%)	4 (1.0)
retroperitoneal bleeding, *n* (%)	8 (2.1)
Blood transfusion, *n* (%)	31 (8.2)
Baseline creatinine level, median (IQR) (g/dL)	107 (87.0–147.6)
Creatinine level after procedures, median (IQR) (g/dL)	104 (91.0–149.2)
Urgent cardiac surgery, *n* (%)	13 (3.5)
Permanent pacemaker implantation, *n* (%)	3 (0.8)
Hospital stay duration, median (IQR) (days)	5.5 (4.0–11.5)
Intraprocedural mortality, *n* (%)	9 (2.4)

BAV—balloon aortic valvuloplasty.

**Table 5:** Cumulative follow-up mortality data.

	All (*n* = 374)
In-hospital mortality, *n* (%)	19 (5.1)
30-day mortality rate, *n* (%)	39 (10.4)
6-month mortality rate, *n* (%)	81 (21.6)
12-month morality rate, *n* (%)	106 (28.3)
12-month mortality in palliative group, *n* (%)	93 (66.9) *

* *n* = 139, number of patients undergoing balloon aortic valvuloplasty as destination therapy.

**Table 6:** Multivariable Cox model for all-cause mortality.

	HR (95% CI)	*p*	HR (95% CI) Adjusted for Age/Gender	*p*
Age	0.87 (0.68–1.15)	0.25	-	-
Sex (female)	0.94 (0.72–2.01)	0.14	-	-
Hypertension	1.12 (0.79–1.79)	0.29	1.05 (0.81–1.63)	0.31
Coronary artery disease	1.06 (0.71–1.56)	0.37	1.03 (0.73–1.43)	0.43
Diabetes	0.82 (0.75–1.34)	0.23	0.83 (0.87–1.29)	0.54
Atrial fibrillation	1.03 (0.86–1.52)	0.30	1.01 (0.90–1.42)	0.47
Cerebrovascular event	1.23 (0.75–2.13)	0.16	1.18 (0.79–2.04)	0.24
Chronic obstructive pulmonary disease	1.19 (0.76–1.99)	0.14	1.14 (0.77–1.87)	0.28
Chronic kidney disease	1.27 (0.80–2.15)	0.19	1.23 (0.78–1.92)	0.32
STS score (per 1%)	1.25 (1.08–2.46)	0.001	1.42 (1.34–2.88)	<0.0001
LVEF < 20% at baseline	1.65 (1.04–2.67)	0.02	1.89 (1.55–2.83)	<0.0001
LVEF < 30% at 1 month	1.87 (1.35–3.43)	0.001	1.97 (1.62–3.67)	<0.0001

LVEF—left ventricle ejection fraction.

## Data Availability

The data presented in this study are available on request from the corresponding author.
